# Longitudinal Analysis of Peripheral and Colonic CD161^+^ CD4^+^ T Cell Dysfunction in Acute HIV-1 Infection and Effects of Early Treatment Initiation

**DOI:** 10.3390/v12121426

**Published:** 2020-12-11

**Authors:** Kerri G. Lal, Yuwadee Phuang-Ngern, Suchada Suhkumvittaya, Edwin Leeansyah, Aljawharah Alrubayyi, Joana Dias, Adam Waickman, Dohoon Kim, Eugène Kroon, Suteeraporn Pinyakorn, Leigh Anne Eller, Milton Maciel Jr., Rungsun Rerknimitr, Nitiya Chomchey, Nittaya Phanuphak, Mark S. de Souza, Sorachai Nitayaphan, Julie A. Ake, Sandhya Vasan, Merlin L. Robb, Jintanat Ananworanich, Johan K. Sandberg, Alexandra Schuetz, Michael A. Eller, Dominic Paquin-Proulx, on behalf of the RV217, RV254/SEARCH010, RV304/SEARCH Study Groups

**Affiliations:** 1 U.S. Military HIV Research Program, Walter Reed Army Institute of Research, Silver Spring, MD 20910, USA; klal@hivresearch.org (K.G.L.); aljawharah.alrubayyi@linacre.ox.ac.uk (A.A.); dkim@hivresearch.org (D.K.); spinyakorn@hivresearch.org (S.P.); leller@hivresearch.org (L.A.E.); jake@hivresearch.org (J.A.A.); SVasan@hivresearch.org (S.V.); MRobb@hjf.org (M.L.R.); jintanat@usthai.us (J.A.); schuetza@hiv-th.org (A.S.); michael.eller@nih.gov (M.A.E.); 2 Henry M. Jackson Foundation for the Advancement of Military Medicine Inc., Bethesda, MD 20817, USA; Milton.macieljr@gmail.com; 3 Center for Infectious Medicine, Department of Medicine, Karolinska Institutet, 14183 Stockholm, Sweden; edwin.leeansyah@ki.se (E.L.); joana.dias15@gmail.com (J.D.); johan.sandberg@ki.se (J.K.S.); 4 Department of Retrovirology, Armed Forces Research Institute of Medical Sciences, Bangkok 10400, Thailand; yuwadeep@hiv-th.org (Y.P.-N.); suchadas@hiv-th.org (S.S.); sorachain.rta@afrims.org (S.N.); 5 Tsinghua-Berkeley Shenzhen Institute, Tsinghua University, Shenzhen 518055, China; 6 Programme in Emerging Infectious Diseases, Duke-National University of Singapore Medical School, Singapore 169857, Singapore; 7 Viral Diseases Branch, Walter Reed Army Institute of Research, Silver Spring, MD 20910, USA; waickmaa@upstate.edu; 8 SEARCH, Institute of HIV Research and Innovation, Bangkok 10330, Thailand; eugene.k@searchthailand.org (E.K.); nitiya.c@searchthailand.org (N.C.); nittaya.p@searchthailand.org (N.P.); 9 Department of Immunology and Microbiology, Uniformed Services University of the Health Sciences, Bethesda, MD 20814, USA; 10 Faculty of Medicine, Chulalongkorn University, Bangkok 10330, Thailand; ercp@live.com; 11 AIDS Research Center, National Institute of Infectious Diseases, Tokyo 162-0052, Japan; markdes@searchthailand.org; 12 Department of Global Health, Academic Medical Centre, Meibergdreef 9, 1105 AZ Amsterdam, The Netherlands; 13 Amsterdam Institute for Global Health and Development, AHTC, Tower C4, Paasheuvelweg 25, 1105 BP Amsterdam, The Netherlands

**Keywords:** HIV-1, CD4, CD161, Th17, IL-12, IL-18

## Abstract

CD161 expression on CD4^+^ T cells is associated with a Th17 functional phenotype, as well as with an innate capacity to respond to interleukin (IL)-12 and IL-18 without T cell receptor (TCR) stimulation. Chronic HIV-1 infection is associated with loss of the CD161^+^ CD4 T cell population, and non-human primate studies suggest that their depletion is associated with disease progression. However, the dynamics of the CD161^+^ CD4^+^ T cell population during acute HIV-1 infection remains unknown. In this study, we characterize peripheral blood CD161^+^ CD4^+^ T cells in detail, and examine how they are affected during the earliest stages of HIV-1 infection. Unbiased surface proteome screening and principal component analysis indicated that CD161^+^ CD4^+^ T cells are relatively phenotypically homogeneous between donors, and are intermediates between conventional CD4 T cells and innate-like T cells. In acute untreated HIV-1 infection, the circulating CD161^+^ CD4^+^ T cell population decreased in frequency, as did absolute cell counts starting from peak viral load, with elevated levels of activation and exhaustion markers expressed throughout acute HIV-1 infection. The capacity of these cells to respond to stimulation with IL-12 and IL-18 was also reduced. Early initiation of anti-retroviral treatment (ART) during acute HIV-1 infection restored the functionality of peripheral blood CD161^+^ CD4^+^ T cells, but not their frequency. In contrast, early ART initiation prevented the decline of colonic CD161^+^ CD4^+^ T cells that otherwise started during acute infection. Furthermore, loss of peripheral and colonic CD161^+^ CD4^+^ T cells in untreated infection was associated with levels of viral load. These results suggest that acute HIV-1 infection has profound effects on the CD161^+^ CD4^+^ T cell population that could not be completely prevented by the initiation of ART.

## 1. Introduction

CD161 is a member of the C-type lectin family, and was originally described as a natural killer (NK) cell receptor; it was subsequently found expressed on subsets of both CD4^+^ and CD8^+^ T cells [1]. CD161 ligation on T cells can provide a co-stimulatory signal for T cell receptor (TCR)-mediated activation [2]. Furthermore, several studies have shown that CD161 expression on T cells is associated with the capacity to produce interleukin (IL)-17 [3,4,5]. Innate-like T cells, such as mucosal associated invariant T (MAIT) [6] and invariant natural killer T (iNKT) [7] cells, are all characterized by CD161 expression. Interestingly, conventional CD8^+^ and CD4^+^ T cells expressing CD161 were reported to share a transcriptional program with innate-like T cells, as well as responsiveness to cytokine stimulation [8]. Furthermore, CD161 expression on CD4^+^ T cells is associated with markers of gut homing receptors, such as C-C chemokine receptor (CCR) 6 and integrin α4β7 [5].

CCR6^+^ CD4^+^ T cells are highly permissive to HIV-1 infection, and are depleted from peripheral blood during chronic HIV-1 infection (CHI) [9]. CD161^+^ CD4^+^ T cells are also depleted from peripheral blood during CHI, and are susceptible to infection by both CCR5 and C-X-C chemokine receptor (CXCR) 4-tropic viruses [10,11]. Furthermore, CD161^+^ CD4^+^ T cells are enriched in the healthy female genital tract, but are depleted in this tissue during CHI [12]. Recently, CD161^+^ CD4^+^ T cells were shown to harbor replication-competent HIV-1 clones in the peripheral blood [11], thus contributing to the latent viral reservoir. In SIV models, the depletion of peripheral CD161^+^ CD4^+^ T cells was linked to disease progression in rhesus macaques, while no depletion was observed in the natural SIV host, sooty mangabeys [13]. The finding of increased levels of CD161^+^ CD4^+^ T cells in the intestines 4 to 6 weeks following SIV infection suggests redistribution of these cells to this tissue. Altogether, these data suggest important roles for CD4^+^ CD161^+^ T cells in HIV-1 infection and disease progression. However, how early these cells are lost after HIV-1 infection is unclear, and the impact of early anti-retroviral treatment (ART) initiation remain unknown. Furthermore, the impact of HIV-1 infection on the function of CD4^+^ CD161^+^ T cells is unknown.

In this study, we investigate CD161^+^ CD4^+^ T cell frequency, phenotype, and function in a longitudinal manner in individuals prior to infection, and in acute HIV-1 infection (AHI) with or without ART. The results suggest that loss of both colonic and peripheral blood CD161^+^ CD4^+^ cells occurs during the earliest stages of HIV-1 infection. Furthermore, CD161^+^ CD4^+^ T cells have an activated and exhausted phenotype, accompanied by a reduced capacity to produce cytokines following IL-12 and IL-18 stimulation during AHI. Reconstitution of colonic, but not peripheral, CD161^+^ CD4^+^ T cells was observed two years following ART initiation during AHI. Overall, our findings indicate that the innate and Th17-like CD161^+^ CD4^+^ T cells are severely affected during acute stages of HIV-1 infection, and that early ART initiation may preserve these cells in the colon.

## 2. Materials and Methods

### 2.1. Study Participants

The RV217 ECHO study has been described previously [14]. Briefly, the RV217 study enrolled consenting adults from key populations at four clinical research sites in Kenya, Uganda, Tanzania, and Thailand. HIV-uninfected participants were screened twice weekly for HIV-1 infection through finger pricks analyzed by a nucleic acid amplification test (NAAT; Aptima HIV-1 RNA Qualitative test, Hologic Inc., San Diego, CA, USA). Enrollees with reactive NAAT were enrolled in a second phase of the study that included intensive sampling of larger blood volumes throughout acute infection and into chronic infection. All HIV-1 positive participants were referred to care providers for management of the infection, based on national guidelines. Treatment was usually available at no cost through host nation care and treatment programs. Eligibility for ART initiation varied over study period and by country, according to recommendations in national guidelines that changed over time. The cases presented in this study are a selected set from a group of 20 RV217 participants for whom cryopreserved peripheral blood mononuclear cells (PBMCs) were available prior to infection, and at least three post-infection time points corresponding to peak viral load (VL) (median days since first positive test for HIV-1 RNA = 16), set point VL (median days since first positive test for HIV-1 RNA = 43), and early chronic infection (median days since first positive test for HIV-1 RNA = 85). Lymphocyte absolute counts were performed real time on whole blood using the Trucount, lyse no wash, Multitest platform (Becton Dickinson Biosciences, San Jose, CA, United States) to enumerate T cell, B cell, and NK cell subsets.

The RV254/SEARCH 010 study is an ongoing acute HIV-1 infection cohort based in Bangkok, Thailand (Clinicaltrials.gov identification: NCT00796146). ART was provided within a few days of diagnosis under a separate protocol (Clinicaltrials.org identification: NCT00796263). Blood samples were screened in real time by pooled NAAT and sequential EIA, according to published methods [15]. Participants who had positive NAAT (confirmed by quantitative HIV-1 RNA) and nonreactive HIV IgG were enrolled in the RV254/SEARCH 010 cohort. Sampling of mucosal biopsies was performed by sigmoidoscopy as an optional study procedure at time of HIV diagnosis (*n* = 26) and two years after ART initiation (*n* = 20). ART was initiated on median 4 days from cohort enrollment. The first seven subjects included in this analysis were treated with standard doses of tenofovir/emtricitabine/efavirenz/raltegravir/maraviroc, while the subsequent subjects were randomized to either this regimen or tenofovir/emtricitabine/efavirenz. Plasma, PBMCs, and mucosal mononuclear cells (MMCs), from HIV-uninfected Thai individuals participating in protocol RV304 (Clinicaltrials.gov identification: NCT01397669) who underwent the same procedures were used as controls. A separate cohort of healthy, HIV-uninfected individuals were recruited at the Blood Transfusion Clinic of Karolinska University Hospital Huddinge.

### 2.2. Study Approval

The RV254/SEARCH 010 and RV304/SEARCH 013 studies (Clinicaltrials.gov identifications: NCT00796146 24 November 2008 and NCT01397669 19 July 2011, respectively) were approved by the Institutional Review Boards (IRBs) of Chulalongkorn University in Thailand and the Walter Reed Army Institute of Research in the United States. Initiation of ART was voluntary under an accompanying protocol (Clinicaltrials.gov identification: NCT00796263 24 November 2008), approved by the Chulalongkorn University IRB. The RV217 study was approved by the Walter Reed Army Institute of Research in the United States and relevant IRBs in Kenya, Uganda, Tanzania, and Thailand. For all studies, subjects gave written informed consent.

### 2.3. Biopsy Processing and Calculation of Absolute Number of Colonic T Cell Subset

Subjects underwent a routine sigmoidoscopy procedure with or without moderate conscious sedation. Approximately 30 endoscopic biopsies were randomly collected from the sigmoid colon using Radial Jaw 3 biopsy forceps (Boston Scientific, Natick, MA, USA), not accounting by visual control for the potential collection of lymphoid aggregates, with 20–25 processed for flow cytometry analysis within 30 min of collection, as previously described [16]. The cell count for all mucosal samples was done manually by trypan blue exclusion, which allows for the exclusion of epithelial cells due to their different morphology compared to lymphocytes. Absolute numbers of CD4^+^ T cells per gram of gut tissue were calculated by multiplying the total viable lymphocyte count by frequencies of cell subsets obtained from flow cytometric analysis. The total lymphocyte count per gram of tissue was calculated by dividing the viable lymphocyte count by the tissue weight. This proportion was then multiplied by the percent of cells in the live lymphocyte gate, and that number was subsequently multiplied by the percent of CD3^+^ lymphocytes. The absolute number of colonic CD3^+^ T cells was used in conjunction with the subset percentages to determine the absolute number of each T cell subset per gram of biopsy tissue.

### 2.4. Flow Cytometry

Frequency and phenotype of peripheral blood and mucosal CD161^+^ CD4^+^ T cells were determined as previously described [17]. Briefly, thawed samples were washed, stained with LIVE/DEAD Fixable Aqua Dead Cell dye (ThermoFisher, Waltham, MA, USA), blocked for Fc receptors using normal mouse serum (ThermoFisher), and surface-stained with an antibody cocktail. Samples were surface-stained at room temperature for 30 min. Surface staining was performed at 37 °C for panels, including CCR5 antibodies. Cells were then washed and fixed in 2% paraformaldehyde. Cells were fixed in Cytofix/Cytoperm or in Transcription Factor Fixation/Permeabilization buffer (both from BD Biosciences, San Jose, CA, USA) as appropriate for transcription factor analysis. Intracellular staining was performed using the relevant mAbs in Perm/Wash or Transcription Factor Perm/Wash buffer as appropriate (both from BD Biosciences). The LEGENDScreen was performed as previously described [18]. Samples were acquired on a five-laser, 16-parameter BD LSRII SORP; an 18-parameter LSR Fortessa; or a four-laser, custom-built LSR Fortessa (all from BD Biosciences). Data were analyzed with FlowJo v.9.9.4 or higher (BD Biosciences). See Supplemental Experimental Procedures for specific antibodies used throughout the study.

### 2.5. Functional Assays

Functional assays were performed as previously described [19]. PBMCs were stimulated for a total of 24 h with IL-12 (10 ng/mL, Peprotech, Rocky Hill, NJ, USA) and IL-18 (100 ng/mL Medical & Biological Laboratories, Nagoya, Japan); monensin (eBioscience, San Diego, CA, USA) and Brefeldin A (BD Biosciences) were added during the last 6 h of stimulation. Data was collected the same day on a 14-color BD LSRII SORP.

### 2.6. Statistical Analysis

All statistical analysis was performed using Graph Pad Prism version 8.2.0 for Mac OS (GraphPad Software, La Jolla, CA, USA). Longitudinal comparisons were performed using the Friedman test. Comparisons between HIV-uninfected and HIV-infected were performed using the Mann–Whitney test. Principal component analysis (PCA) was performed with the R basic function, prcomp method, and the results were visualized using R plotly bioconductor package. Associations were evaluated using Spearman’s rank correlation; *p*-values <0.05 were considered statistically significant.

## 3. Results

### 3.1. CD161^+^ CD4^+^ T Cells Express RORγt and Produce Th1 and Th17 Cytokines in Response to IL-12 and IL-18 Stimulation

**Figure 1:**
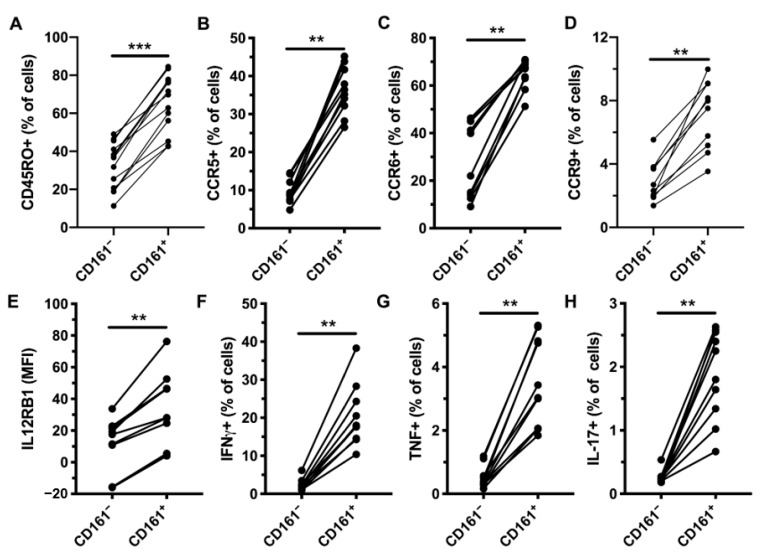

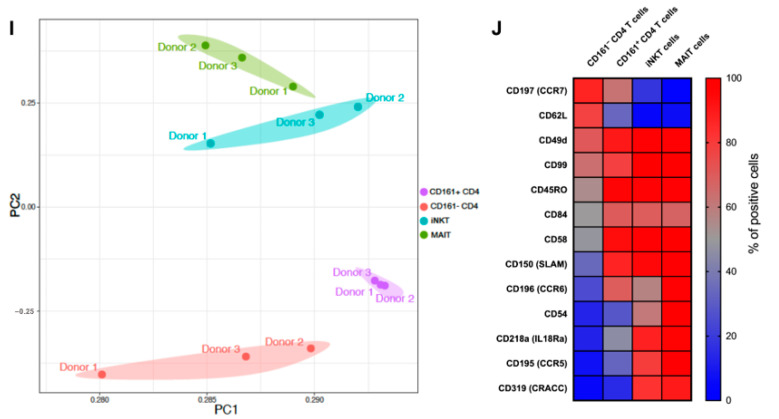
Phenotype and function of peripheral blood CD161^+^ CD4^+^ T cells in HIV-uninfected subjects. Levels of CD45RO (**A**, *n* = 13), CCR5 (**B**, *n* = 10), CCR6 (**C**, *n* = 10), CCR9 (**D**, *n* = 10), and IL-12RB1 (**E**, *n* = 10) on CD161^−^ and CD161^+^ CD4^+^ T cells. Production of IFNγ (**F**), TNF (**G**), and IL-17 (**H**) by CD161^−^ and CD161^+^ CD4^+^ T cells following stimulation with IL-12 and IL-18 for 24 h (*n* = 10). Principal component analysis (PCA) of the surface proteome dataset, with the four T cell populations plotted against principal component (PC) 1 and PC2 (**I**). Heat map showing the average expression levels of selected markers for the four T cell populations (**J**). ** *p* < 0.01, and *** *p* < 0.001.

Normal, healthy donor CD161^+^ CD4^+^ T cells were phenotyped using flow cytometry, excluding known innate T cells, namely MAIT (Vα7.2^+^ CD161^+^) and iNKT (Vα24^+^ Vβ11^+^) cells (Supplementary Figure S1A). As expected, CD161^+^ CD4^+^ T cells had higher levels of CD45RO, CCR5, CCR6, CCR9, and IL-12Rβ1 (Figure 1A–E and Supplementary Figure S1B–F) than their CD161^−^ counterparts. Next, we evaluated the capacity of these cells to respond to IL-12 and IL-18 stimulation, as measured by IFNγ production (Figure 1F and Supplementary Figure S1G). CD161^+^ CD4^+^ T cells also produced TNF and IL-17 following cytokine stimulation (Figure 1G,H and Supplementary Figure S1G). We performed an extensive characterization of CD161^+^ CD4^+^ T cells in relation to CD161^−^ CD4^+^ and other innate T cells by screening three healthy donors by flow cytometry, using a panel of 332 immuno-receptors. PCA revealed that CD161^+^ CD4^+^ T cells were relatively phenotypically homogenous. CD161^+^ CD4^+^ T cells had an intermediate profile in between conventional CD4 T cells and the innate-like iNKT and MAIT cells (Figure 1I). The shared pattern of surface marker expression between CD161^+^ CD4^+^ T cells and innate-like T cells included higher levels of CD49d (integrin α4), CD99, CD45RO, CD84, CD58, SLAM, CCR6, CD54, IL-18Rα, CCR5, and CRACC, as well as lower levels of CCR7 and CD62L compared to CD161^−^ CD4^+^ T cells (Figure 1J), with the expression level of CD161^+^ CD4^+^ T cells often being in between their CD161^−^ counterpart and innate-like T cells. These results are consistent with previous studies that have identified CD161^+^ CD4^+^ T cells as containing Th17 cells and sharing an innate program allowing them to respond to cytokine stimulation independent of TCR engagement [9].

**Figure 2:**
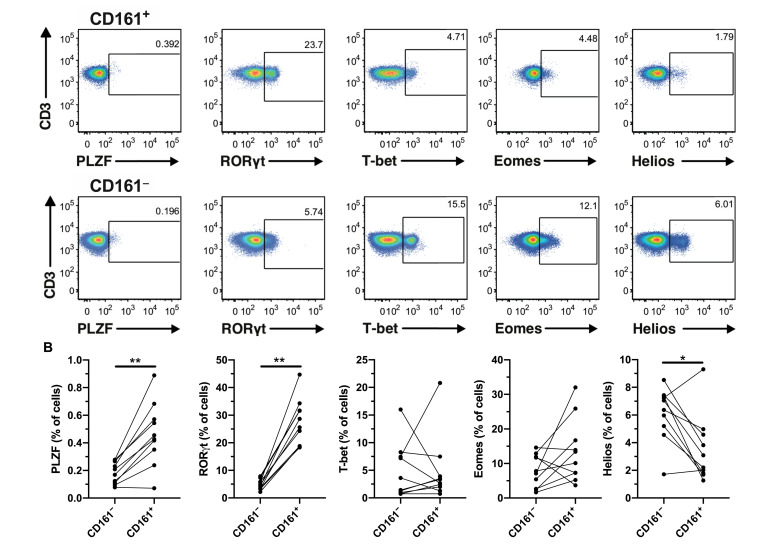
Transcription factors expressed by CD161^+^ CD4^+^ T cells. Representative flow plots showing expression of RORγt, PLZF, Helios, Eomes, and T-bet in CD161^+^ and CD161^−^ CD4^+^ T cells (**A**). Level of PLZF, RORγt, T-bet, Eomes, and Helios in CD161^+^ and CD161^−^ CD4^+^ T cells (*n* = 10, **B**). * *p* < 0.05 and ** *p* < 0.01.

We next investigated expression of the transcription factors RAR-related orphan receptor (ROR) γt, promyelocytic leukemia zinc finger (PLZF), Helios, eomesodermin (Eomes), and T-box expressed in T cells (T-bet) in CD161^+^ and CD161^−^ CD4 T cells (Figure 2A). CD161^+^ cells had significantly higher levels of RORγt compared to CD161^−^ CD4 T cells, consistent with a Th17 profile (Figure 2B). PLZF expression was also higher in CD161^+^ cells, although the levels were low. Helios, a transcription factor important for regulatory T cells [20], was significantly lower in CD161^+^ cells. No differences in the levels of Eomes and T-bet were detected.

### 3.2. CD161^+^ CD4^+^ T Cells Have a Diverse TCR Repertoire

Next, in an effort to further characterize the diversity of CD161^+^ CD4^+^ T cells, we compared their TCR repertoire to their CD161^−^ counterparts. Single-cell RNA sequencing was performed on PBMCs from eight HIV-negative healthy donors. All immune cell populations could be identified by unsupervised clustering (Figure S2A,B). As expected, CD161 (*KLRB1*)-relative gene expression was high in MAIT cells and NK cells, and could also be detected in memory CD4^+^ T cells (Figure S2C). Memory CD4^+^ T cells were then grouped into *KLRB1* positive and negative cells (Figure S2D) for TCR repertoire analysis. The Shannon diversity index, a statistical method used to determine the relative diversity of species between groups, was performed to determine if CD161^−^ and CD161^+^ CD4^+^ T cells have comparably diverse TCR repertoires. CD161^+^ CD4^+^ T cells had a slightly lower diversity score compared to CD161^−^ CD4^+^ T cells for both TCR alpha (6.13 vs. 6.50) and beta (5.42 vs. 5.62) chains (Figure S2E,F). Together, this suggests that *KLRB1* high CD4^+^ T cells display a TCR repertoire diversity that overall is similar to the main memory CD4^+^ T cell pool.

### 3.3. CD161^+^ CD4^+^ T Cells Decline in Peripheral Blood During AHI, Have an Activated Phenotype and Decreased Functional Capacity

**Figure 3:**
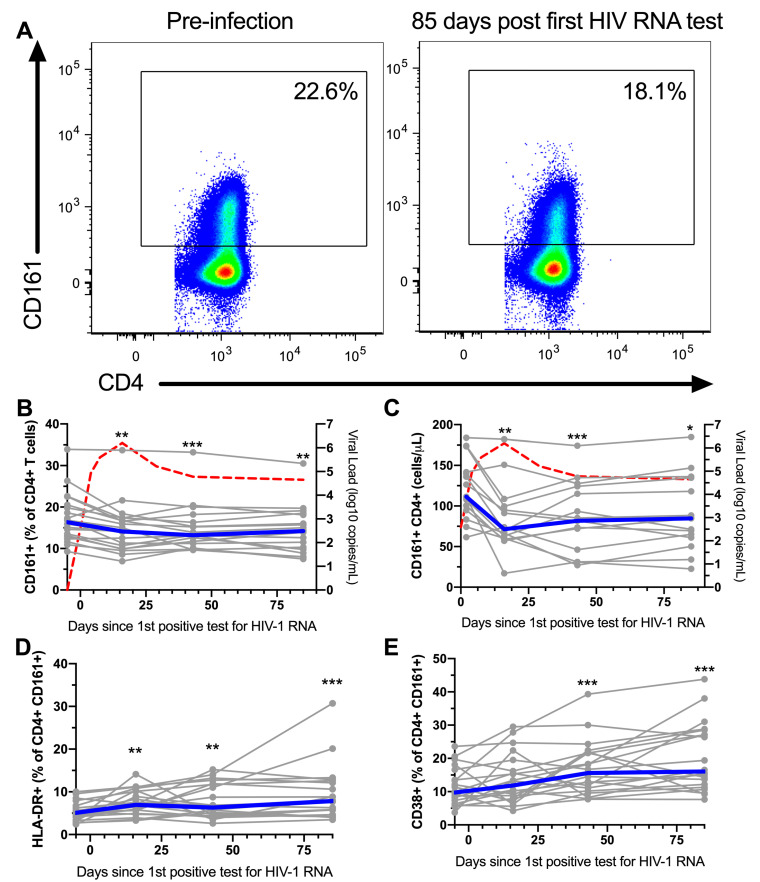

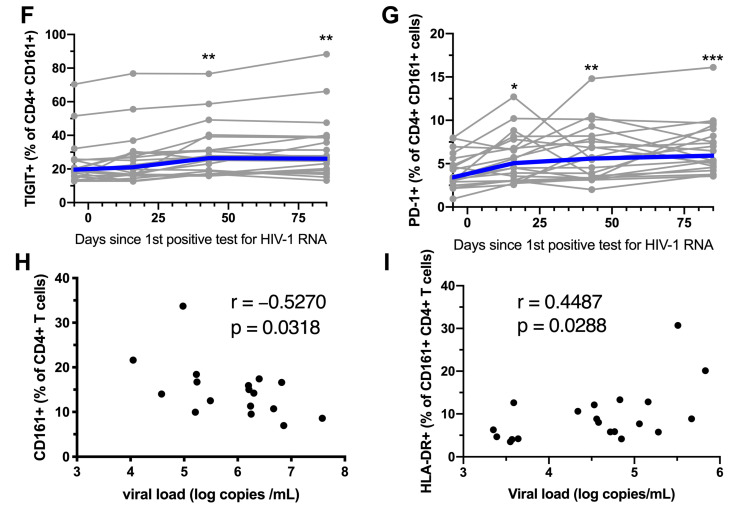
Peripheral blood CD161^+^ CD4^+^ T cells are reduced early in HIV-1 infection. Representative flow plots showing expression of CD161 by CD4^+^ T cells in blood in acute, untreated HIV-1 infection (**A**). Frequency (**B**) and absolute cell count (**C**) of peripheral blood CD4^+^ T cells expressing CD161 during untreated, acute HIV-1 infection (AHI). Individual subjects are shown in grey and the median in blue. Time points sampled are indicated by the circles. The red line represents the median viral load. Absolute cell count of peripheral blood cells in acute untreated HIV-1 infection. Expression of HLA-DR (**D**), CD38 (**E**), TIGIT (**F**), and PD-1 (**G**), by peripheral blood CD161^+^ CD4^+^ T cells in acute, untreated HIV-1 infection. Association between VL 16 days post-first HIV RNA test, and frequency of peripheral CD4^+^ T cells expressing CD161 (**H**). Associations between VL at day 85 post-first HIV RNA-positive test and expression of HLA-DR by CD161^+^ CD4^+^ T cells (**I**). *n* = 20 for all plots except for (**C**) (*n* = 15) and (**H**) (*n* = 17). * *p* < 0.05, ** *p* < 0.01, and *** *p* < 0.001.

**Figure 4:**
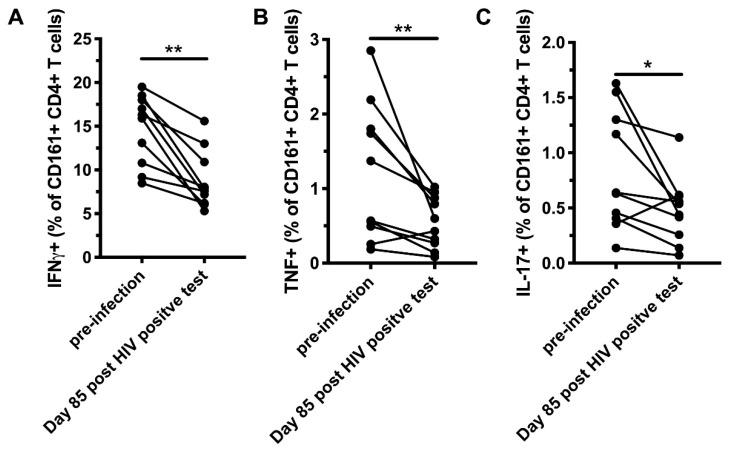
Reduced cytokine production by peripheral blood CD161^+^ CD4^+^ T cells during early chronic HIV-1 infection. Production of IFNγ (**A**), TNF (**B**), and IL-17 (**C**) by CD161^+^ CD4^+^ T cells before and 85 days after the first HIV RNA-positive test, following stimulation with IL-12 and IL-18 for 24 h (*n* = 10). * *p* < 0.05 and ** *p* < 0.01.

As CD161^+^ CD4^+^ T cells are memory cells, expressing high levels of CCR5, CCR6, and integrin α4, a phenotype associated with susceptibility to HIV-1 infection, we studied how these cells are affected during untreated AHI. PBMCs from 20 subjects with donor-matched samples corresponding to pre-infection, peak HIV-1 VL, viral set point, and early chronic infection (corresponding to a median 16, 43, and 85 days after the first HIV-1 RNA positive test, respectively) from the HIV ECHO (RV217) cohort from East Africa and Thailand [14] (Table 1) were analyzed to determine the frequency and phenotype of peripheral blood CD161^+^ CD4^+^ T cells (Figure 3A and Supplementary Figure S3). The frequency of CD161^+^ cells within the CD4^+^ T compartment was reduced at the first available time point post-infection in AHI, corresponding to peak HIV-1 VL, and at all subsequent time points measured compared to pre-infection (Figure 3B). For 15 of those participants, longitudinal absolute cell counts were available starting from day 2 post-first HIV-1 RNA positive test. Similar to frequency, absolute CD161^+^ CD4^+^ cell counts were reduced in peripheral blood during AHI (Figure 3C). We next evaluated the levels of the activation markers HLA-DR and CD38, as well as exhaustion markers T cell immunoreceptor with Ig and ITAM domains (TIGIT) and programmed cell death protein 1 (PD-1) on CD161^+^ CD4^+^ T cells throughout the course of AHI (Supplementary Figure S3). HLA-DR expression was elevated starting from peak VL, and remained elevated at the subsequent time points compared to pre-infection (Figure 3D). Starting from viral set point, CD38 was significantly elevated and remained elevated through early chronic infection (Figure 3E). TIGIT and PD-1 levels were also increased starting from viral set point and peak VL, respectively (Figure 3F,G). Next, we investigated if perturbations in the CD161^+^ CD4^+^ T cell compartment were associated with HIV-1 replication. At the time point corresponding to peak VL, there was an inverse association between VL and the frequency of CD161^+^ cells within the CD4^+^ T cell compartment (rho = −0.53, *p* = 0.03, Figure 3H). Furthermore, there was a significant positive association between VL and levels of HLA-DR expression (rho = 0.49, *p* = 0.03) at day 85 post-first HIV RNA positive test, corresponding to early chronic infection (Figure 3I). No significant associations were found with between VL and the expression of CD38, TIGIT, and PD-1 levels on CD161^+^ CD4^+^ T cells. To assess if HIV-1 infection impacts the functional response of CD161^+^ CD4^+^ T cells, PBMCs from before the infection and day 85 post-first HIV RNA positive test were stimulated with IL-12 and IL-18, and the production of cytokines was evaluated by flow cytometry. The capacity of CD161^+^ CD4^+^ T cells to produce IFNγ, TNF, and IL-17 was decreased post-HIV-1 infection compared to pre-infection (Figure 4). These results suggest that the CD161^+^ CD4^+^ T cell population is significantly altered during untreated AHI.

### 3.4. Pre-Infection Levels of CCR5^+^ CD161^+^ CD4^+^ T Cells are Inversely Associated with CD4 Nadir

We then investigated if the characteristics of CD161^+^ CD4^+^ T cells pre-infection were associated with HIV acquisition or disease progression. The frequency of CD161^+^ CD4^+^ T cells at enrollment in the 20 individuals that became HIV-1 infected was compared with that of 19 community-matched individuals that did not become HIV-1 infected during the course of the RV217 study. Surprisingly, individuals that remained uninfected had higher levels of CD161^+^ CD4^+^ T cells within the total T cell compartment, compared to pre-infection levels in individuals that eventually acquired HIV (Figure S4A). In contrast, individuals that acquired HIV had a significant inverse association between the pre-infection levels of CD4 T cells co-expressing CCR5 and CD161 pre-infection and the CD4 nadir (rho = −0.68, *p* = 0.04; Figure S4B). There were no such associations with CCR5^+^ CD161^−^ cells (rho = −0.22, *p* = 0.54; data not shown), or with peak VL and VL set points.

### 3.5. ART Initiation During AHI Restores the Phenotype and Functional Response, but not Frequency of Peripheral Blood CD161^+^ CD4^+^ T Cells

**Figure 5:**
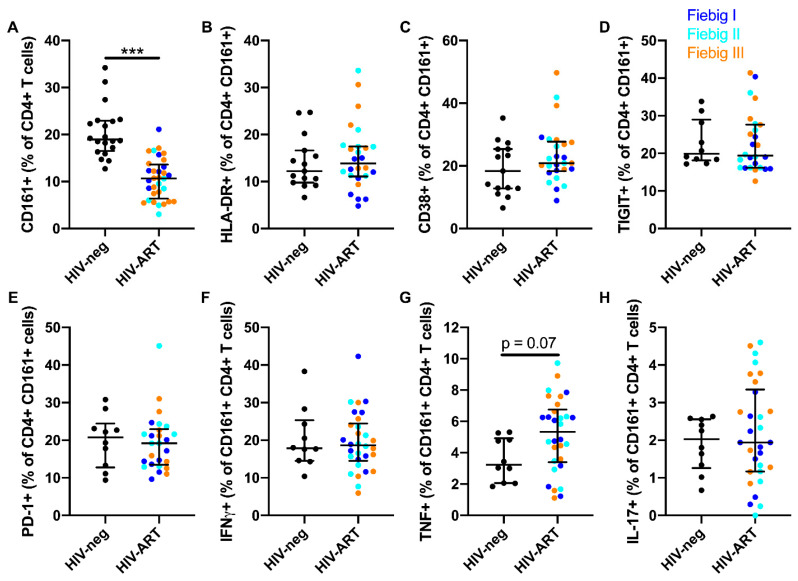
ART initiation during AHI restores normal phenotype and functions, but not the frequency of peripheral CD161^+^ CD4^+^ T cells. Frequency of peripheral CD161^+^ CD4^+^ T cells in HIV-uninfected (*n* = 20) and HIV-infected individuals that initiated ART during Fiebig stage I (*n* = 8), Fiebig stage II (*n* = 7), and Fiebig stage III (*n* = 17) (**A**). Levels of HLA-DR (**B**), CD38 (**C**), TIGIT (**D**), and PD-1 (**E**) expression by CD161^+^ CD4^+^ T cells in HIV-uninfected (*n* = 15 for **B** and **C** and *n* = 10 for **D** and **E**) and HIV-infected individuals that initiated ART during Fiebig stage I (*n* = 9), Fiebig stage II (*n* = 8), and Fiebig stage III (*n* = 17). Production of IFNγ (**F**), TNF (**G**), and IL-17 (**H**) by CD161^+^ CD4^+^ T cells following IL-12 and IL-18 stimulation in HIV-uninfected (*n* = 10) and HIV-infected individuals that initiated ART during Fiebig stage I (*n* = 9), Fiebig stage II (*n* = 8), and Fiebig stage III (*n* = 17). The lines and whiskers represent the median and interquartile range, respectively. *** *p* < 0.001.

Because a previous study reported that the frequency of CD161^+^ CD4^+^ T cells remains reduced when ART is initiated during CHI [10], we investigated if ART initiation during AHI could prevent the decline of these cells. For this purpose, we analyzed samples from the RV254/SEARCH010 cohort, wherein ART is initiated during AHI [21]. Community-matched, HIV-1-uninfected Thai individuals were used as a control group (Table 2). The proportion of peripheral blood CD4^+^ T cells expressing CD161 remained reduced after two years of suppressive therapy in acutely treated individuals (Figure 5A). However, expression of the activation markers HLA-DR and CD38, as well as the exhaustion markers TIGIT and PD-1, were similar to those of HIV-uninfected subjects (Figure 5B–E). Next, we evaluated if early ART initiation could prevent the functional decline of CD161^+^ CD4^+^ T cells. Peripheral CD161^+^ CD4^+^ T cells from acutely treated subject had a similar capacity to produce interferon gamma (IFNγ), tumor necrosis factor (TNF), and IL-17 after stimulation with IL-12 and IL-18, compared to HIV-uninfected individuals (Figure 5F–H). This suggests that early ART prevents phenotypic and functional perturbations in CD161^+^ CD4^+^ T cells, but fails to restore their frequency in peripheral blood within the early chronic infection stage.

### 3.6. Early ART Initiation Restores CD161^+^ CD4^+^ T Cells in the Colonic Mucosa

**Figure 6:**
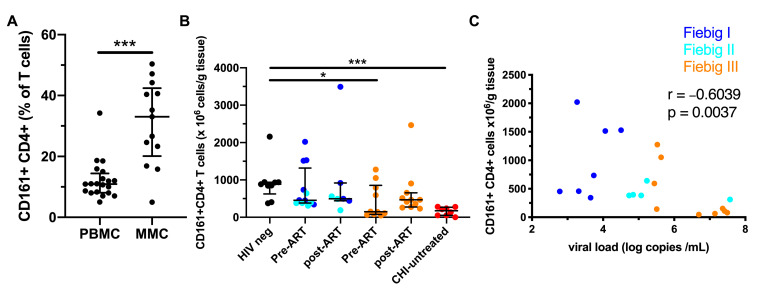
Depletion of CD161^+^ CD4^+^ T cells in the colonic mucosa is prevented by early ART. Frequency of CD161^+^ CD4^+^ T cells in peripheral blood (*n* = 20) and colonic mucosa (*n* = 13) of HIV-uninfected Thai subjects (**A**). Absolute number of colonic CD161^+^ CD4^+^ T cells in HIV-uninfected subjects (black, *n* = 9), Fiebig I (dark blue) Fiebig II (light blue), and Fiebig III (orange) HIV-infected individuals before ART (*n* = 22) and after two years of ART initiated during AHI (*n* = 18) (**B**). Association between VL and absolute number of colonic CD161^+^ CD4^+^ T cells during AHI (**C**). The lines and whiskers represent the median and interquartile range, respectively. *** *p* < 0.001 and * *p* < 0.05.

The colonic mucosa is an important site of HIV replication [22,23], and we and others have shown that CD161^+^ CD4^+^ T cells express high levels of molecules associated with gut homing [5]. Thus, we studied the dynamics of colonic CD161^+^ CD4^+^ T cells during AHI and following early ART initiation. CD161^+^ was expressed on approximately one-third of the CD4^+^ T cells in the colonic mucosa in uninfected subjects, a proportion significantly higher than in peripheral blood (Figure 6A). In AHI, colonic CD161^+^ CD4^+^ T cells were depleted beginning in Fiebig stage III (Figure 6B). Following two years of treatment initiated during AHI, CD161^+^ CD4^+^ T cell levels were similar to those in HIV-uninfected individuals. The abundance of CD161^+^ CD4^+^ T cells in the colonic mucosa during AHI was inversely associated with VL (rho = −0.60, *p* = 0.004; Figure 6C). This suggests that ART initiation during early AHI is associated with the restoration of CD161^+^ CD4^+^ T cells in the colonic mucosa.

## 4. Discussion

Here, we elucidate the dynamics of CD161^+^ CD4^+^ T cells in HIV-1 infection. We excluded MAIT and iNKT cells from our analysis, as these cells represent very defined subsets of T cells with distinct properties. In this study, CD161^+^ CD4^+^ T cells were confirmed to share some features with innate-like T cells, including the capacity to produce IFNγ following IL-12 and IL-18 stimulation. Furthermore, these cells produced low levels of IL-17 in response to innate cytokine stimulation, consistent with their expression of RORγt and previous studies identifying these cells as having a Th17 profile [3,5]. The ability of these cells to produce IL-17 may be more pronounced in the tissue or proinflammatory environments, as in the case with other innate-like T cell populations, which is an area of research to be further explored [24,25,26,27]. CD161^+^ CD4^+^ T cell shared features with the typical innate-like T cells, also including a memory phenotype and a pattern of chemokine receptor expression. Studies using RNAseq have identified a gradient in the effectorness [28] and innateness [29] in T cells. Similarly, our surface proteome analysis indicates that CD161^+^ CD4^+^ T cells cluster in between MAIT/iNKT cells and CD161^−^ CD4^+^ T cells, with expression levels of key receptors associated with “innateness” (IL18Ra, CCR7) following this pattern. Unlike MAIT cells and iNKT cells, CD161^+^ CD4^+^ T cells have a diverse TCR repertoire. We have identified several chemokine receptors that are differently expressed by CD161^+^ CD4^+^ T cells and their CD161^−^ counterparts, suggesting an important difference in tissue homing and trafficking associated with CD161 expression. Furthermore, we found enrichment of these cells in the colonic mucosa in HIV-uninfected individuals. Overall, the CD161^+^ CD4^+^ T cells share many characteristics with previously described, antigen-independent memory CD4^+^ T cells [30].

The colonic mucosa is an important site for viral replication during AHI and CD161^+^ CD4^+^ T cells, expressing higher levels of the HIV coreceptor CCR5 compared to their CD161^−^ counterpart. We have also found higher expression of CCR6 on CD161^+^ CD4^+^ T cells, and it has been reported that HIV-1 selectively targets gut-homing CCR6^+^ cells [31]. Moreover, CD161^+^ CD4^+^ T cells have been reported to express the integrin α4β7 [5], and our surface proteome analysis confirmed higher expression of α4 and β7 by CD161^+^ CD4^+^ T cells. Integrin α4γ7 can bind the HIV-1 envelope glycoprotein, and several studies suggest that α4β7^+^ CD4 T cells are preferentially infected during AHI [32,33,34,35]. Collectively, these observations point towards the ability to be directly infected with HIV-1, and suggest a possible mechanism behind the depletion of CD161^+^ CD4^+^ T cells in the peripheral blood and colonic mucosa during AHI. This is supported by the inverse association between VL and the frequency of CD161^+^ CD4^+^ T cells in both peripheral blood and colonic mucosa in the present study, as well as previous studies, indicating their high susceptibility to HIV-1 infection in vitro [10,11]. However, in the ECHO cohort, pre-infection frequency of CD161^+^ CD4^+^ T cells in the peripheral blood were higher in participants that remained uninfected during the course of the study, compared to those that became HIV-infected. Confirmation of this finding and determining the cause for this observation will require more investigation, including studies with a higher number of individuals. One possibility is that lower levels of peripheral CD161^+^ CD4^+^ T cells are associated with inflammation in mucosal compartments that could increase the risk of acquiring HIV-1.

Our findings indicate that HIV-1 infection is associated with a reduced capacity of CD161^+^ CD4^+^ T cells to produce cytokines following stimulation with IL-12 and IL-18. Concurrent levels of the exhaustion markers PD-1 and TIGIT were elevated, suggesting that the CD161^+^ CD4^+^ T cells are already exhausted during the course of HIV-1 infection as early as the viral set point, approximately one month post-infection. Early ART initiation restored the functionality of CD161^+^ CD4^+^ T cells and normalized their levels of activation and exhaustion markers. The frequency of these cells was restored in the colonic mucosa, but not in peripheral blood upon ART initiation in the acute infection stages. One possible explanation for this phenomenon is that residual inflammation in the mucosa is driving the recruitment of these cells to tissues. In fact, initiation of ART during AHI does not fully normalize levels of immune activation [36], and such recruitment of CD161^+^ CD4^+^ T cells to the colonic mucosa has been suggested in untreated SIV infection [13]. Significant depletion of CD161^+^ CD4^+^ T cells in the colonic mucosa occurred from Fiebig stage III, consistent with our previous observations regarding Th17 cells [16]. One limitation of our study is that the HIV-uninfected groups had a higher proportion of females. The impact of gender on CD161^+^ CD4^+^ T cells frequency is unknown, but in our cohort of HIV-uninfected participants, we did not find a difference in the frequency of CD4^+^ T cell expressing CD161 (data not shown).

Our data, together with others, suggest that CD161^+^ CD4^+^ T cells are comprised to a large extent of Th17 cells. Th17 cells are believed to play an important antimicrobial role in the tissue, and may be involved in maintaining mucosal integrity [37,38,39,40]. In immunosuppressed cancer patients or transplantation recipients, a low frequency of CD161^+^ CD4^+^ T cells has been associated with increased risk of infections [41,42]. In mice, memory phenotype CD4 T cells producing IFNγ in response to IL-12, a similar characteristic to what we have described in humans for CD161^+^ CD4^+^ T cells, provide resistance against *Toxoplasma gondii* infection in an antigen-independent manner by enhancing adaptive Th1 responses [43]. Therefore, the lack of complete recovery of CD161^+^ CD4^+^ T cells in peripheral blood, despite initiation of ART at the earliest possible stage, could contribute to the susceptibility to bacterial infections seen in ART-treated individuals [44,45,46]. Future immunotherapies that aim to restore these cells to normal levels in peripheral blood could be beneficial to reducing HIV-associated comorbidities. However, CD161^+^ CD4^+^ T cells have also been shown to harbor replication-competent HIV-1 clones [11], and therefore careful consideration should be taken to restore these cells without expanding viral reservoirs.

In summary, we have provided further evidence that CD161^+^ CD4^+^ T cells share some characteristics with innate-like T cells, and shown that these cells are depleted early during AHI, both in peripheral blood and the colonic mucosa. The residual CD161^+^ CD4^+^ T cell population has an activated/exhausted phenotype, and a reduced functional capacity following innate cytokine stimulation. Early initiation of ART restores a normal phenotype and functional capacity of these cells in circulation, but their frequency remained reduced. In contrast, early ART prevents the loss of colonic CD161^+^ CD4^+^ T cells.

## Figures and Tables

**Table 1:** Acute untreated (RV217) subjects’ demographics.

Characteristics	Acute HIV-Infected (*n* = 22)	HIV-Uninfected (*n* = 20)
Median age (years)	23 (18, 35) ^A^	25 (18, 45) ^A^
Gender, Male/Female/TGW	8:9:5	2:17:1
Country, *n* (%)		
Uganda	6 (27.4)	5 (20)
Tanzania	3 (13.6)	5 (20)
Kenya	2 (9)	5 (20)
Thailand	11 (50)	5 (20)
Median CD4^+^ T cell nadir (cells/mL)	483 (286, 866) ^A^	NA
Median time to peak VL (days)	14 (6, 19) ^A^	NA
Median peak VL (log_10_ copies/mL)	6.68 (5.49, 7.94) ^A^	NA
Median set point VL (log_10_ copies/mL)	4.46 (3.52, 5.96) ^A^	NA

^A^ Range; CD4^+^ T cell nadir = minimum CD4^+^ T cell count prior to day 80; set point viral load (VL) = average of all measured VL between day 80 and day 365, in the absence of treatment (required at least two measurements); TGW = transgender women; NA: not applicable.

**Table 2:** Clinical, immunological, and virological characteristics and demographics of RV254/SEARCH 010 and RV304/SEARCH 013 study participants.

Characteristics	Acute HIV-Infected at Time of Diagnosis (*n* = 23)	Acute HIV-Infected Post-ART-Initiation (*n* = 40)	HIV-Uninfected (*n* = 28)
Median age (years)	29 (19, 46) ^A^	27 (18, 54) ^A^	34 (20–43) ^A^
Gender, Male:Female:TGW	21:2:0	39:1:0	14:6:4
Risk behavior, *n* (%)			
MSM	19 (82.6)	34 (85)	14 (50)
Bisexual male	2 (8.7)	1 (2.5)	-
Heterosexual male	-	4 (10.0)	4 (14.3)
Heterosexual female	2 (8.7)	1 (2.5)	6 (21.4)
TGW	-	-	4 (14.3)
Fiebig Stage, *n*			
I/II	12 (7 I, 5 II)	24 (11 I, 13 II)	NA
III	11	16	NA
Mean (SD) days since HIV exposure to enrollment	16.3 (5.7)	17.0 (7.2)	NA
Mean (SD) time to ART initiation following diagnosis (days)	NA	4 (1.7)	NA
Median duration of ART (weeks)	NA	12 (12–96) ^A^	NA
Median plasma HIV RNA (log_10_ copies/mL)	5.4 (2.8, 7.7) ^A^	1.6 (1.3, 2.6) ^A^	NA
Median sigmoid colon HIV RNA (log_10_ copies/mg tissue)	3.1 (1.3, 6.1) ^A^	1.7 (1.7, 1.7) ^A^	NA
Median CD4^+^ T cell count (cell/mm^3)^	532 (132, 1127) ^A^	890 (452, 1,266) ^A^	1005 (738–2059) ^A^

^A^ Range; MSM: men who have sex with men; TGW: transgender woman; Fiebig I: positive HIV RNA, negative p24 antigen, negative third-generation EIA; Fiebig II: positive HIV RNA, positive p24 antigen, negative third-generation EIA; Fiebig III: positive HIV RNA, positive p24 antigen, positive third-generation EIA, negative Western blot; NA: Not Applicable.

## Supplementary Materials

The following are available online at https://www.mdpi.com/1999-4915/12/12/1426/s1, Supplementary Methods, Figure S1: Representative flow plots for the phenotype and functional analysis of CD161^+^ CD4^+^ T cells, Figure S2: Cellular identification and KLRB1 gene expression analysis of healthy donor PBMC by scRNAseq, Figure S3: Expression of activation and exhaustion markers by CD161^+^ CD4^+^ T cells, Figure S4: Pre-infection levels of CD161^+^ CD4^+^ T cells associations with HIV-1 acquisition and disease progression.

## Disclaimer

The content of this manuscript is solely the responsibility of the authors, and does not necessarily represent the official views of any of the institutions mentioned above, the U.S. Department of the Army or the U.S. Department of Defense, the Henry M. Jackson Foundation for the Advancement of Military Medicine, the National Institutes of Health, the Department of Health and Human Services, or the United States government, nor does mention of trade names, commercial products, or organizations imply endorsement by the Thai Red Cross AIDS Research Centre. The investigators have adhered to the policies for protection of human participants as prescribed in AR-70-25.

## Abbreviations

TCR	T cell receptor
IL	Interleukin
CCR	C-C chemokine receptor
CXCR	C-X-C chemokine receptor
NAAT	Nucleic acid amplification test
IRBs	Institutional Review Boards
PBMCs	Peripheral blood mononuclear cells
MMCs	Mucosal mononuclear cells
ROR	RAR-related orphan receptor
PLZF	Promyelocytic leukemia zinc finger
Eomes	Eomesodermin
T-bet	T-box expressed in T cells
TIGIT	T cell immunoreceptor with Ig and ITAM domains
PD-1	Programmed cell death protein 1
IFNγ	Interferon gamma
TNF	Tumor necrosis factor
MAIT	Mucosal-associated invariant T
iNKT	Invariant natural killer T
CHI	Chronic HIV-1 infection
AHI	Acute HIV-1 infection
ART	Anti-retroviral treatment
VL	Viral load
PCA	Principal Component Analysis
