# Supplementary material for: Longitudinal Analysis of Peripheral and Colonic CD161+ CD4+ T Cell Dysfunction in Acute HIV-1 Infection and Effects of Early Treatment Initiation
Source: Viruses. 2020 Dec 11;12(12):1426. doi: 10.3390/v12121426 (PMC7764746; doi:10.3390/v12121426)
Supplement: Supplementary file 1 [file viruses-12-01426-s001.pdf]

Supplementary Methods

CD161$^+$ CD4$^+$ T cell frequency, activation, phenotype, and flow cytometry panels. Briefly, thawed samples were washed, stained with LIVE/DEAD Fixable Aqua Dead Cell dye (ThermoFisher), blocked for Fc receptors using normal mouse serum (ThermoFisher), and surface stained with antibody cocktail. 20 donors with longitudinal samples from one pre-infection and three post-infection time points corresponding to peak VL, set point VL, and early chronic infection were surface stained. Additional individuals with pre-infection time points only were stained following the same protocol. The phenotyping and surface panel included TCR Vα24 FITC (clone C15, Beckman Coulter), TCR Vβ11 PE (clone C21, Beckman Coulter), CD14 AlexaFluor700 (clone M5E2, BD Biosciences), CD19 AlexaFluor700 (clone H1B19, BD Biosciences), CD3 PE Texas Red (clone S4.1, ThermoFisher), CD4 APC-H7 (clone SK3, BD Biosciences), CD8 PerCP-Cy5.5 (clone SK1, BD Biosciences), HLA-DR BV650 (clone L243, BioLegend), CD38 BV711 (clone HIT2, BioLegend), CD45RO BV786 (clone UCHL1, BD Biosciences), CD161 PE-Cy (clone DX12, BD Bioscience) or BV 605 (clone HP-3G10, Biolegend), PD-1 BV785 (clone EH12.1, Biosciences), TIGIT PE-Cy7 (clone MBSA43, ThermoFisher), IL12RB1 BV786 (clone 2.4E6, BD Biosciences), CCR5 BUV737 or APC-Cy7 (clone 2D7/CCR5, BD Biosciences), CCR6 BV650 or PE-Cy7 (clone 11A9, BD Biosciences), CCR9 PE-Cy7 (clone L053E8, Biolegend), RORγt PE (clone Q21-559, BD Bioscience), Eomes FITC (clone WD1928, Ebioscience), Helios eFluor 450 (clone 22F6, Ebioscience), T-bet BV605 or BV711 (clone 4B10, Biolegend), PLZF APC (clone 6318100, R&D systems).

Biopsy processing and flow cytometry. Subjects underwent a routine sigmoidoscopy procedure under moderate conscious sedation. Approximately 25-30 endoscopic biopsies were randomly collected from the sigmoid colon using Radial Jaw 3 biopsy forceps (Boston Scientific, Natick, MA, USA) and processed for flow cytometry analysis within 30 min of collection. In groups of five the biopsies were weighed and placed in 500 ml of RPMI media containing 10% human AB serum (HAB; Gemini Bio-Product, West Sacramento,

CA, USA), 1% HEPES, 1% L-Glutamine, 0.1% Gentamicin (Invitrogen, Carlsbad, CA, USA), 1% Penicillin/Streptomycin and 2.5 mg/ml Amphotericin B (Invitrogen, Carlsbad, CA, USA). Samples were then digested using 0.5 mg/ml Collagenase II (Sigma, St. Louis, MO, USA). After digestion samples were filtered through a cell strainer, using a syringe with a 16-gauge blunt end needle. This procedure was repeated once or twice in case undigested tissue remained. After being washed twice with RPMI containing 1% HEPES, 1% L-Glutamin, 1% Penicillin/Streptomycin, 0.1% Gentamycin and 2.5 mg/ml Amphotericin B, MMC were counted and viable cell enumeration was determined using Trypan Blue exclusion and Beckman Coulter AcT5 hematology analyzer (Beckman Coulter, Fullerton, CA). Freshly isolated MMC were stained with LIVE/DEAD Fixable Aqua Dead Cell dye, washed, surface stained with TCR Vα24 FITC, TCR Vβ11 PE, CD14 AlexaFluor700 (clone M5E2, BD Pharmingen), CD19 AlexaFluor700 (clone HIB19, BD Pharmingen), CD3 APC-H7 (clone SK7, BD Pharmingen), CD4 ECD (clone SFCI12T4D11, Beckman Coulter), CD8 V450 (clone RPA-T8, BD Horizon), fixed with 1% paraformaldehyde, and data was acquired on a 4-laser custom-built LSR Fortessa (BD Biosciences).

Functional assay. PBMC were stained for living cells with Aqua LIVE/DEAD viability dye (ThermoFisher), Fc receptors were blocked with normal mouse serum (ThermoFisher), and surface stained for 30 minutes at room temperature. After fixation with 2% paraformaldehyde, iNKT cells were stained for intracellular cytokines for 30 minutes with Perm/Wash Buffer (BD Biosciences). Anti-human antibodies used to stain for intracellular cytokines included IFNγ BV785 (clone 4S.B3, BioLegend), TNF BV650 (clone MAb11, BD Biosciences), IL-17 BV711 (clone BL168, Biolegend).

Single-cell RNA sequencing library generation: Thawed PBMC suspensions were prepared for single-cell RNA sequencing using the Chromium Single-Cell 5′ Reagent version 2 kit and Chromium Single-Cell Controller (10x Genomics, CA)[1]. 2000–8000 cells per reaction suspended at a density of 50–500 cells/μL in PBS plus 0.5% FBS were loaded for gel bead-in-emulsion (GEM) generation and barcoding. Reverse

transcription, RT-cleanup, and cDNA amplification were performed to isolate and amplify cDNA for downstream 5′ gene or enriched V(D)J library construction according to the manufacturer's protocol. Libraries were constructed using the Chromium Single-Cell 5′ reagent kit, V(D)J Human B Cell Enrichment Kit, 3′/5′ Library Construction Kit, and i7 Multiplex Kit (10x Genomics, CA) according to the manufacturer's protocol.

Sequencing: scRNAseq 5′ gene expression libraries and BCR V(D)J enriched libraries were sequenced on an Illumina NovaSeq 6000 instrument using the S2, or S4 reagent kits (300 cycles). Libraries were balanced to allow for ~150,000 reads/cell for 5′ gene expression libraries, and ~20,000 reads/cell for BCR V(D)J enriched libraries. Sequencing parameters were set for 150 cycles for Read1, 8 cycles for Index1, and 150 cycles for Read2. Prior to sequencing, library quality and concentration were assessed using an Agilent 4200 TapeStation with High Sensitivity D5000 ScreenTape Assay and Qubit Fluorometer (Thermo Fisher Scientific) with dsDNA BR assay kit according to the manufacturer's recommendations

5' gene expression analysis/visualization: 5′ gene expression alignment from all PBMC samples was performed using the 10x Genomics Cell Ranger pipeline[1]. Sample demultiplexing, alignment, barcode/UMI filtering, and duplicate compression was performed using the Cell Ranger software package (10x Genomics, CA, v2.1.0) and bcl2fastq2 (Illumina, CA, v2.20) according to the manufacturer's recommendations, using the default settings and mkfastq/count commands, respectively. Transcript alignment was performed against a human reference library generated using the Cell Ranger mkref command and the Ensembl GRCh38 v87 top-level genome FASTA and the corresponding Ensembl v87 gene GTF.

Multi-sample integration, data normalization, dimensional reduction, visualization, and differential gene expression was performed using the R package Seurat (v3.1.4)[2,3]. All datasets were filtered to only contain cells with between 200-6,000 unique features and <10% mitochondrial RNA content. To eliminate

erythrocyte contamination, datasets were additionally filtered to contain cells with less than a 5% erythrocytic gene signature (defined as HBA1, HBA2, HBB). Data were scaled, transformed, and variable genes identified using the SCTransform() function. SelectIntegrationFeatures() and PrepSCTIntegration() functions were used to identify conserved features for dataset integration, and final dataset anchoring/integration performed using FindIntegrationAnchors() and IntegrateData() functions, with the day 0 DHIM samples and day 180 natural infection samples used as reference datasets. PCA was performed using variable genes defined by SCTransform() additionally filtered to remove TCR V/D/J or BCR k/l gene segments, as well as mitochondrial associated genes (^MT) and ribosomal proteins (^RP). The first 30 resultant PCs were used to perform a UMAP dimensional reduction of the dataset (RunUMAP()) and to construct a shared nearest neighbor graph (SNN; FindNeighbors()). This SNN was used to cluster the dataset (FindClusters()) with default parameters and resolution set to 0.5.

Following dataset integration and dimensional reduction/clustering, gene expression data was log_e(UMI+1) transformed and scaled by a factor of 10,000 using the NormalizeData() function. This normalized gene expression data was used to determine cellular cluster identity by utilizing the Seurat application of a Wilcoxon rank-sum test (FindAllMarkers(), and comparing the resulting differential expression data to known cell-linage specific gene sets.

TCR sequence analysis: TCR clonotype identification, alignment, and annotation was performed using the 10x Genomics Cell Ranger pipeline. Sample demultiplexing and clonotype alignment was performed using the Cell Ranger software package (10x Genomics, CA, v2.1.0) and bcl2fastq2 (Illumina, CA, v2.20) according to the manufacturer's recommendations, using the default settings and mkfastq/vdj commands, respectively. TCR clonotype alignment was performed against a filtered human V(D)J reference library generated using the Cell Ranger mkvdjref command and the Ensembl GRCh38 v87 top-level genome FASTA and the corresponding Ensembl v87 gene GTF. Shannon diversity index was calculated has previously described[4].

Supplementary Figures

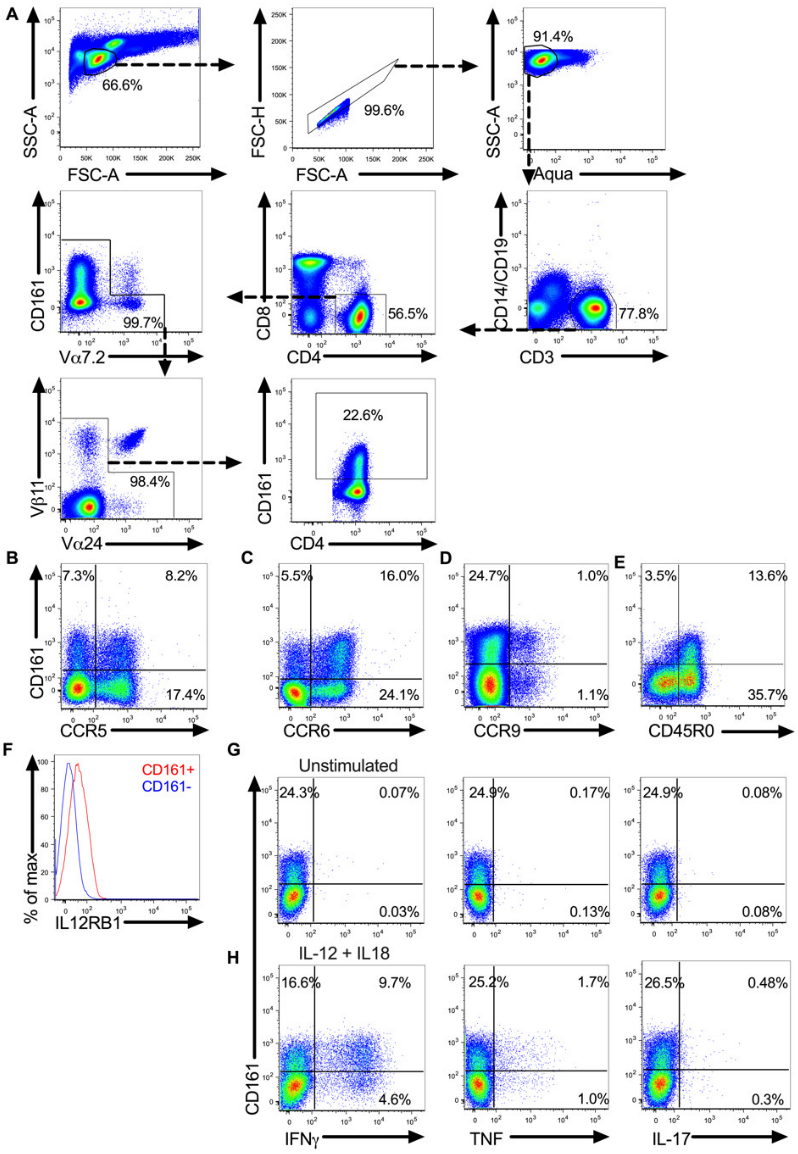

Sup. Figure 1. Representative flow plots for the phenotype and functional analysis of CD161$^+$ CD4$^+$ T cells. Representative flow plots showing the gating strategy for identification of CD161$^+$ CD4$^+$ T cells (A). Representative flow plots showing CCR5 (B), CCR6 (C), CCR9 (D), CD45RO (E), and IL12RB1 (F) levels on CD161$^-$ and CD161$^+$ CD4$^+$ T cells. Representative flow plots showing production of IFNγ, TNF, and IL-17 by unstimulated (G) and IL-12 and IL-18 stimulated (H) CD161$^-$ and CD161$^+$ T for 24 hours.

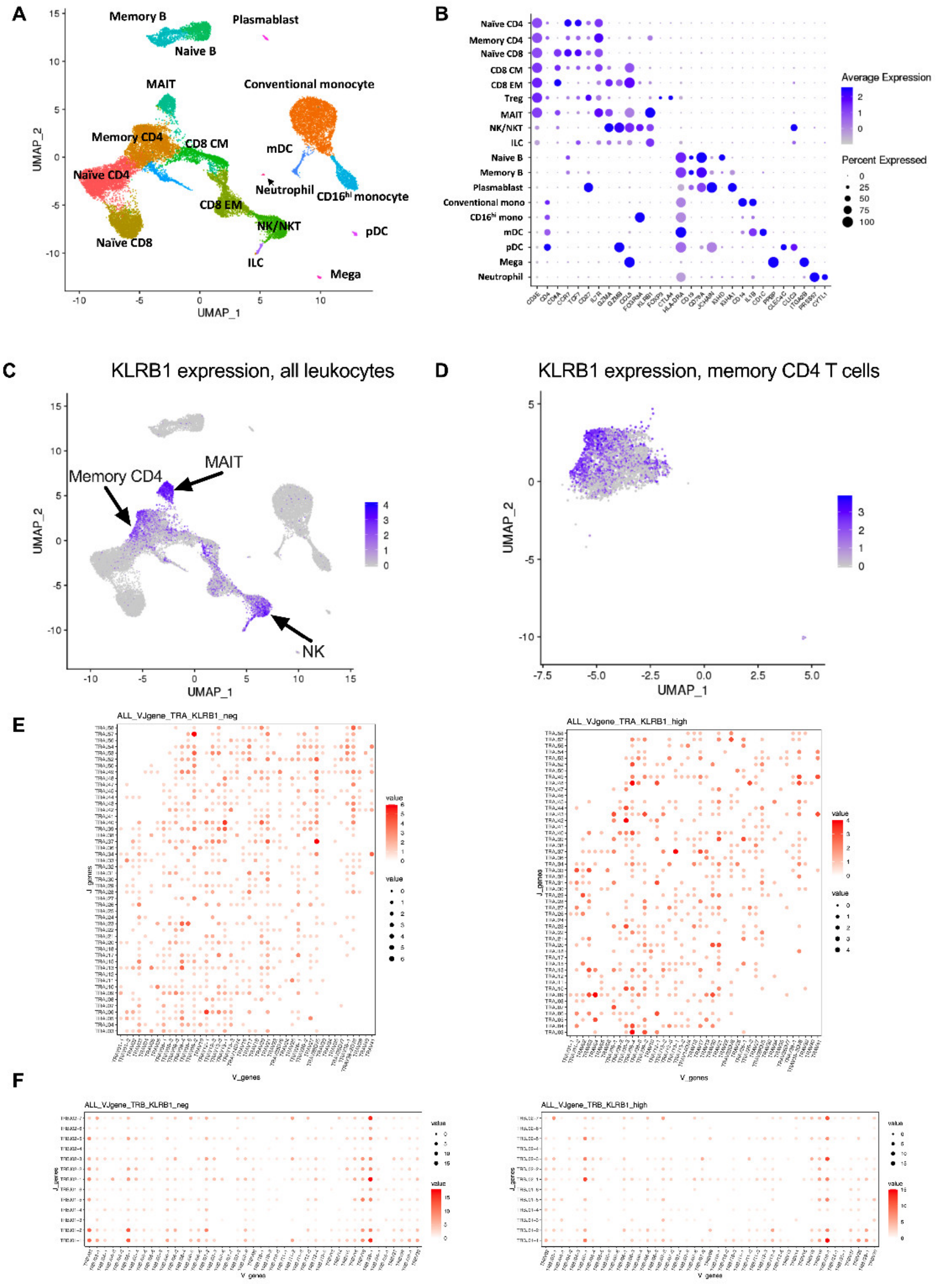

Sup. Figure 2. Cellular identification and KLRB1 gene expression analysis of healthy donor PBMC by scRNAseq. Integrated UMAP projection of 24,973 cells captured by scRNAseq analysis of PBMC obtained from 8 heathy normal donors (A). Expression of key linage defining gene products across all annotated leukocyte populations captured in this analysis (B). UMAP projection of normalized KLRB1 expression across all captured leukocytes (C). UMAP projection of normalized KLRB1 expression across

annotated memory CD4+ T cells (n = 3,625) captured in this analysis (D). Dot plot of paired TCR alpha chain V and J segment usage in KLRB1 negative (left) and KLRB1 positive (right) CD4+ memory cells as assessed by single cell RNA sequencing (E). Frequency of paired chain usage indicated by dot color and size. Dot plot of paired TCR beta chain V and J segment usage in KLRB1 negative (left) and KLRB1 positive (right) CD4+ memory cells as assessed by single cell RNA sequencing (F). Frequency of paired chain usage indicated by dot color and size.

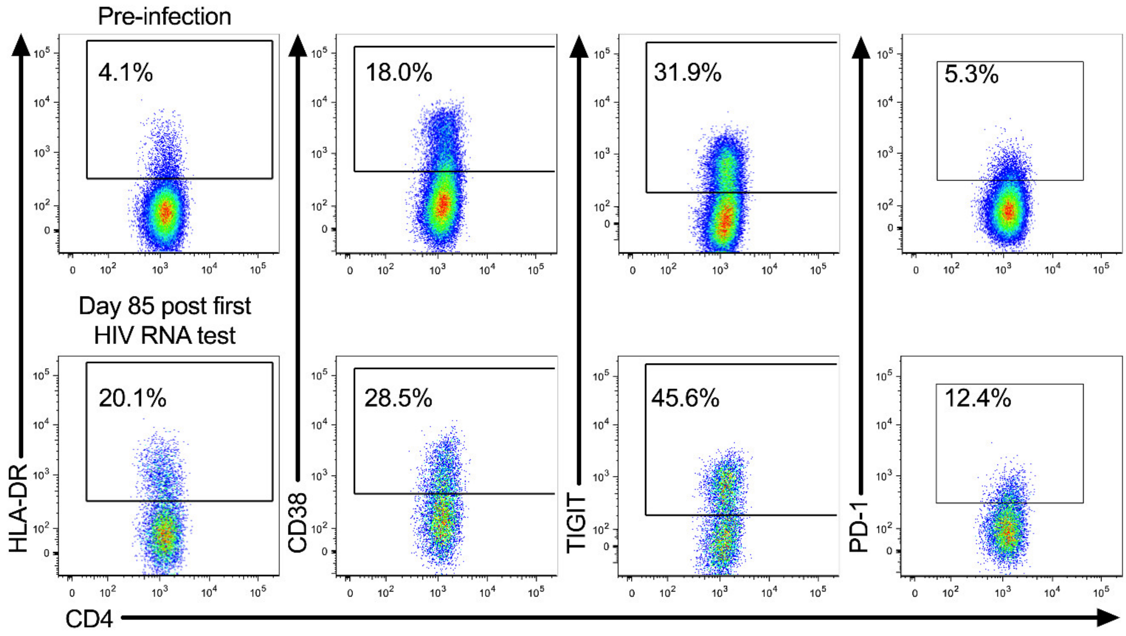

Sup. Figure 3. Expression of activation and exhaustion markers by CD161+ CD4+ T cells. Representative flow plots showing expression of HLA-DR, CD38, TIGIT, and PD-1 before and 85 post HIV RNA positive test.

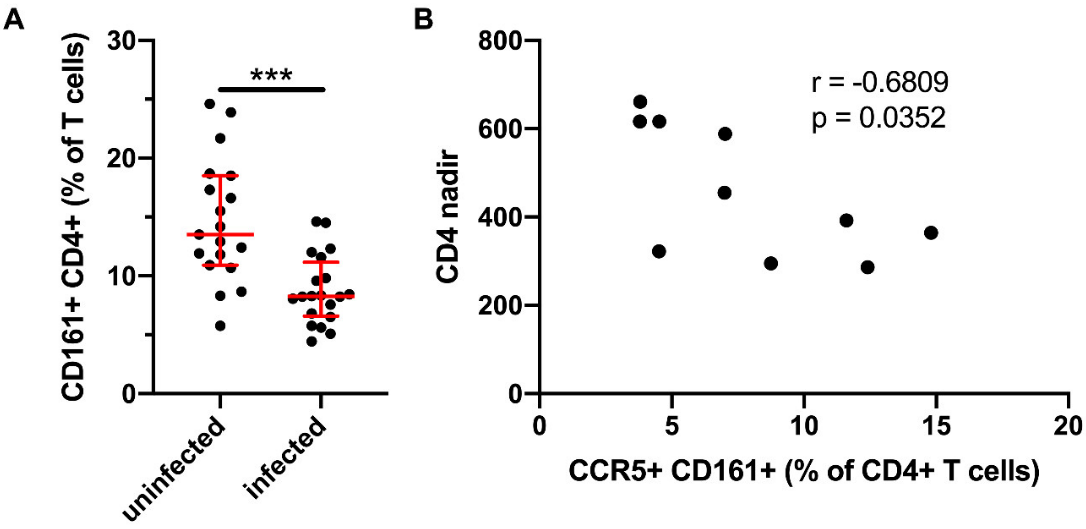

Sup Figure 4. Pre-infection levels of CD161⁺ CD4⁺ T cells associations with HIV-1 acquisition and disease progression. Levels of $CD161^{+}$ $CD4^{+}$ T cells within the total T cell compartment at study enrollment in subjects that remained uninfected (n=19) or became HIV-1 infected (n=20, A). Associations between co-expression of CCR5 and CD161 by CD4 T cells pre-infection and CD4 nadir (n=10, B). *** indicates $p < 0.001$.

References

1. Zheng, G.X.; Terry, J.M.; Belgrader, P.; Ryvkin, P.; Bent, Z.W.; Wilson, R.; Ziraldo, S.B.; Wheeler, T.D.; McDermott, G.P.; Zhu, J., et al. Massively parallel digital transcriptional profiling of single cells. *Nat Commun* **2017**, *8*, 14049, doi:10.1038/ncomms14049.
2. Stuart, T.; Butler, A.; Hoffman, P.; Hafemeister, C.; Papalexi, E.; Mauck, W.M., 3rd; Hao, Y.; Stoeckius, M.; Smibert, P.; Satija, R. Comprehensive Integration of Single-Cell Data. *Cell* **2019**, *177*, 1888-1902 e1821, doi:10.1016/j.cell.2019.05.031.
3. Butler, A.; Hoffman, P.; Smibert, P.; Papalexi, E.; Satija, R. Integrating single-cell transcriptomic data across different conditions, technologies, and species. *Nat Biotechnol* **2018**, *36*, 411-420, doi:10.1038/nbt.4096.
4. Schneider-Hohendorf, T.; Mohan, H.; Bien, C.G.; Breuer, J.; Becker, A.; Gorlich, D.; Kuhlmann, T.; Widman, G.; Herich, S.; Elpers, C., et al. CD8(+) T-cell pathogenicity in Rasmussen encephalitis elucidated by large-scale T-cell receptor sequencing. *Nat Commun* **2016**, *7*, 11153, doi:10.1038/ncomms11153.
